# Breastfeeding, Mother–Child Dyads of Interaction, and Neurodevelopment of Preterm Children: A Longitudinal Study of Feeding Methods During the First Two Years

**DOI:** 10.3390/children12111480

**Published:** 2025-11-02

**Authors:** Júlia Vicente Hass, Carolina Panceri, Rita C. Silveira, Nadia Cristina Valentini

**Affiliations:** 1 Escola de Educação Física, Fisioterapia e Dança (ESEFID), Universidade Federal do Rio Grande do Sul (UFRGS), Porto Alegre 90690-200, Brazil; juliavicentehass@gmail.com; 2 Hospital de Clínicas de Porto Alegre (HCPA), Universidade Federal do Rio Grande do Sul (UFRGS), Porto Alegre 90035-903, Brazil; cpanceri@hcpa.edu.br (C.P.); drarita.c.s@gmail.com (R.C.S.)

**Keywords:** child development, prematurity, breastfeeding, children formula

## Abstract

**Highlights:**

**What are the main findings?**

- Exclusive breastfeeding was associated with consistently higher motor, cognitive, language, and social scores in preterm children during the first two years of life compared to formula feeding; mixed feeding showed intermediate outcomes.
- The quality of mother–child interactions was a stronger predictor of better developmental trajectories, while clinical vulnerabilities (e.g., prolonged NICU stay, BPD, leukomalacia) negatively influenced outcomes.

**What are the implications of the main findings?**

- Breastfeeding functions as both a biological and relational protective factor, supporting neurodevelopment while strengthening sensitive mother–child interactions.
- Interventions that promote breastfeeding and responsive caregiving are crucial strategies to buffer clinical risks and optimize developmental outcomes in preterm children.

**Abstract:**

Objective: Our objectives were as follows: (1) to compare cognitive, motor, language, and social performance, parenting skills, and mother–child interactions among preterm children exposed to different feeding practices (exclusive breastfeeding, mixed feeding, and formula feeding) during the first two years of life; and (2) to examine the associations between the feeding type, risks and protective factors, and neurodevelopmental outcomes (cognitive, motor, language, and social domains) in these population in the first two years of life. Method: A total of 116 preterm children (<32 weeks gestational age—GA) and their mothers were recruited from a public hospital in southern Brazil and followed at a neonatal clinic. Children were organized into three groups based on feeding at NICU discharge (exclusive breastfeeding, formula, or mixed feeding). Assessments were conducted at 4, 8, 12, 18, and 24 months of corrected age using the BSITD-III and additional validated instruments measuring environment stimulation, daily activities, maternal knowledge, breastfeeding experience, and mother–child interactions. Results: Clinical and sociodemographic factors were similar across feeding groups. Group comparisons showed that exclusive breastfeeding was associated with higher motor scores at 12 (*p* = 0.015) and 24 (*p* = 0.026) months, higher cognitive scores at 12 (*p* = 0.049) and 18 (*p* = 0.013) months, and higher language scores at 12 (*p* < 0.001) and 18 (*p* = 0.014) months compared to formula feeding. In the social domain, the exclusive breastfeeding group consistently outperformed the formula group across most time points, although a temporary dip was observed at 18 months. Mixed feeding showed intermediate patterns. Children in the exclusive breastfeeding group also demonstrated higher maternal parenting skills and stronger mother–child interactions across time. Longitudinal analyses revealed that the quality of mother–child interactions predicted better motor, cognitive, language, and social outcomes, while clinical vulnerabilities (e.g., prolonged NICU stay, BPD, leukomalacia) negatively influenced trajectories. Conclusions: Exclusive breastfeeding and responsive mother–child interactions emerged as consistent protective factors across developmental domains, buffering the adverse effects of clinical risks. Breastfeeding not only enhances maternal engagement but also contributes to more favorable motor, cognitive, language, and social outcomes in preterm children. Supporting breastfeeding and promoting sensitive caregiving practices are essential strategies for optimizing developmental trajectories and improving the quality of life in this vulnerable population.

## 1. Introduction

In Brazil, more than 12% of births occur before 37 weeks of gestation [1]. Despite advances in the survival of preterm children [2], many face neurodevelopmental delays, often associated with multiple risk factors [2]. Prematurity and low birth weight are recognized conditions that increase the likelihood of developmental impairments due to the immaturity of organs and systems at birth. These children are more vulnerable to cognitive difficulties, school problems, behavioral changes, and general developmental challenges [3]. In addition to biological risks, environmental factors such as parental education, family income, and the environment physical quality can also significantly influence these outcomes [4]. Recent studies indicate that environmental opportunities and caregivers’ factors can also mitigate previously identified clinical damage, playing a significant role in shaping developmental trajectories [4,5,6,7]. Specifically, for preterm children, sensitive and responsive caregivers play an even more crucial role, creating environmental opportunities that support development [8].

To better understand these complex interactions, this study is grounded in Bronfenbrenner’s bioecological theory of human development [9,10]. This framework highlights that development emerges from dynamic, reciprocal processes between the child and multiple layers of the environment (microsystem, mesosystem, exosystem, macrosystem), evolving over time (chronosystem). For preterm children, biological vulnerabilities interact with proximal processes such as breastfeeding and mother–child interactions, as well as with more distal influences like maternal education, family resources, and health care access. This perspective allows us to integrate clinical, familial, and social factors, clarifying how protective and risk elements shape developmental trajectories during early childhood.

Among environmental factors, breastfeeding offers a well-established immunological benefit, but its impact extends beyond child health—exclusive or even partial breastfeeding enhances child development [11,12]. Previous studies show that human milk contains oligosaccharides and long-chain polyunsaturated fatty acids, which are essential for myelination, neural connectivity, and cognitive development [12,13]. Clinical trials have indicated that children who received formulas enriched with milk fat globule membrane—rich in phospholipids and sphingomyelin—improved myelination in motor-related areas (motor cortices, internal capsule, and cerebellum) and improved nonverbal and fine motor scores [14,15]. Furthermore, breastfeeding reduces the incidence of sepsis, necrotizing enterocolitis, retinopathy of prematurity, and allergies [13,14,16,17].

During the breastfeeding process, high-quality and complex dynamic interactions occur between the mother and child involving maternal sensitivity, responsiveness, and emotional availability—crucial for fostering neurodevelopment outcomes [6,12]. In preterm children, who are often exposed to multiple risk factors, breastfeeding can serve as a key protective factor during periods of high neural plasticity, compensating for biological and clinical vulnerabilities [4,12]. Consequently, exclusive breastfeeding has been recommended during the first six months of life, followed by continued breastfeeding until at least the first year or longer [18]. However, factors such as lack of time, lack of knowledge, emotional difficulties, and an absence of social support can hinder breastfeeding practices [19]. For mothers of preterm children, these difficulties can be intensified due to the newborns’ fragility and the stressful environment of the NICU, which can delay or prevent direct breastfeeding [17,19,20,21].

Despite evidence associating breastfeeding and responsive caregiving to improved outcomes [12,17], there is still a lack of longitudinal studies addressing how these factors interact with clinical risks to influence the motor, cognitive, language, and social development of preterm children in low- and middle-income contexts. Furthermore, although research suggests that preterm children fed with breast milk show better language outcomes [22] and motor development [2], there is a need to better understand how different feeding methods (exclusive breastfeeding, mixed feeding, and formula) relate to both developmental outcomes and parenting quality during the critical early years, specifically longitudinally. It is important to understand how, over time, breastfeeding and mother–child interactions are associated with motor, cognitive, language, and social development in preterm children, considering the multiple interactions that influence these developmental trajectories.

Thus, this study aims to advance the understanding of these protective factors, with the following objectives: (1) to compare cognitive, motor, language, and social performance, parenting skills, and mother–child interactions among preterm children exposed to different feeding practices (exclusive breastfeeding, mixed feeding, and formula feeding) during the first two years of life; and (2) to examine the associations between feeding type, risks and protective factors, and neurodevelopmental outcomes (cognitive, motor, language, and social domains) in these population in the first two years of life. The hypotheses are as follows: (1) Preterm children fed with breast milk (exclusive or mixed) will show higher motor, cognitive, language, and social scores compared to those exclusively formula-fed. (2) Mothers who breastfeed (exclusively or partially) will exhibit stronger parenting skills and more positive mother–child dyads of interactions. (3) Adverse clinical factors will be negatively associated with neurodevelopment outcomes over the first two years of life. (4) Both breastfeeding and the high-quality mother–child dyads of interactions will be positively associated with neurodevelopment outcomes over the first two years of life.

## 2. Materials and Methods

### 2.1. Participants

This was a longitudinal observational study with a quantitative, causal approach. It followed a cohort of preterm children born at Hospital de Clinicas de Porto Alegre, academically linked to the Federal University of Rio Grande do Sul, in southern Brazil, monitored through a neonatal follow-up clinic. The study investigated children exposed to different feeding practices: exclusive breastfeeding, mixed feeding, and formula feeding. The sample included 116 preterm children (gestational age < 32 weeks) and their mothers, drawn from an original cohort of approximately 300. Children born before 32 weeks of gestational age who completed all assessment points at 4, 8, 12, 18, and 24 months of corrected age were included in the study. Exclusion criteria included those born at or beyond 32 weeks of gestation, as well as children with congenital malformations, genetic syndromes, or neurological conditions (e.g., cerebral palsy, hypoxic–ischemic encephalopathy) that could directly affect neurodevelopment, or with incomplete developmental or interaction data. After discharge from the NICU, 26 children (22.4%) were exclusively breastfed, 44 (37.9%) received formula, and 46 (39.7%) received mixed feeding. Most mothers had completed high school (42.2%), and most families had a monthly income around two Brazilian minimum wages, reflecting economic hardship. Families lived in low-income neighborhoods in a major metropolitan area in southern Brazil and received care at the public hospital’s outpatient services. The study was approved by the hospital and university Ethics Committees (Protocol No. 20190321; 2019). Written informed consent was obtained from all participants, and families were free to withdraw at any time.

### 2.2. Instruments

Medical records were used to collect neonatal and sociodemographic data. The neonatal information included gestational age, birth measurements, APGAR scores, duration of NICU stay, bronchopulmonary dysplasia (BPD), small for gestational age (SGA), mechanical ventilation and continuous positive airway pressure (CPAP), parenteral nutrition, sepsis, periventricular hemorrhage, leukomalacia, and type of children feeding. Child development was assessed using Bayley Scales of Children Development, Third Edition (BSID-III), which evaluate cognitive, motor, and language development. For BSID-III results, the raw scale scores are converted into composite scores that are transformed into performance categorizations. Trained professionals following standardized procedures, with scores categorized by developmental level, conducted the assessments. The Daily Activities of Children Scale (DAIS) questionnaire was used to assess how caregivers provided opportunities for children to experience postural control and movement exploration during daily routines. Parental understanding of child development milestones was assessed using the Knowledge of Children Development Inventory (KIDI) scale. The Affordances in the Home Environment for Motor Development–Children Scale (AHEMD-IS) measured environmental support for motor development at home across five domains, including indoor/outdoor space, stimulation variety, and motor materials. The Interaction Rating Scale (IRS) was used to assess child social skills, caregiver behaviors, and mother–child interactions. Finally, maternal perceptions of breastfeeding were assessed with the Breastfeeding Evaluation Scale (MBFES), a 30-item Likert scale evaluating satisfaction, perceived children growth, and impact on maternal lifestyle.

### 2.3. Procedures

While hospitalized, children participated in Kangaroo Care and lactation support programs. After discharge, all children were referred to a neonatal outpatient clinic and followed regularly until 24 months of corrected age. Multidisciplinary assessments occurred monthly until 6 months, bimonthly until 12 months, and quarterly thereafter. BSID-III assessments were conducted at 4, 8, 12, 18, and 24 months in a controlled, child-friendly setting by trained professionals (inter-rater agreement > 96%). Mother–child interactions were recorded during 10 min structured play sessions prior to BSID-III administration. Observers trained in IRS coding rated the interactions (inter-rater agreement > 93%). Parental sociodemographic information was collected via questionnaire at enrollment.

### 2.4. Data Analysis

Continuous variables were expressed as mean and standard deviation, and group differences were assessed using analysis of variance (ANOVA). Categorical variables were presented as frequencies and percentages, and associations between groups were examined with the Chi-Square Test. Normality was tested using Kolmogorov–Smirnov. Repeated measures MANOVA was used to compare developmental and interaction outcomes across feeding groups over time. Pearson correlation and backward linear regression assessed associations. Statistical significance was set at *p* ≤ 0.05. Analyses were performed using SPSS 20.0.

## 3. Results

### 3.1. Clinical and Environmental Outcomes: Group Comparisons

Table 1 presents a comparison of clinical outcomes across time by groups (exclusive breastfeeding, mixed feeding, and formula feeding). Neonatal clinical outcomes were similar across groups (*p*-values ranging from 0.248 to 0.561).

Table 2 presents a comparison of environmental factors among the families of preterm children in the three groups. No significant differences were found across groups. The only exception was in the home affordances assessment: children in the mixed feeding group had a higher number of fine motor toys available at home, followed by the formula group, and the exclusive breastfeeding group.

### 3.2. Cognitive, Motor, Language, and Social Development: Group Comparisons

Table 3 presents means and standard deviations as well as the post hoc tests for motor, cognitive, language, and social development assessed over time by groups.

Regarding motor development, a non-significant group by time interaction was found (Roys’s Largest Root = 0.71, F(4,110) = 1.97, *p* = 0.104, η^2^ = 0.07, Power = 0.58). The group (F(2,113) = 3.34, *p* = 0.039, η^2^ = 0.06, Power = 0.62) and time main effects (F(4,110) = 4.70, *p* = 0.002, η^2^ = 0.15, Power = 0.94) were significant. Post hoc tests showed that the exclusive breastfeeding group had higher scores than the mixed and formula groups at 12 months, and higher scores than the formula group at 24 months. Regarding changes over time, the post hoc test showed that the formula group had decreases in motor scores, whereas the other two groups did not.

Regarding cognitive development, a significant group by time interaction was found (Roy’s Largest Root = 0.10, F(4,110) = 2.83, *p* = 0.028, η^2^ = 0.09, Power = 0.76). The group (F(2,113) = 3.61, *p* = 0.030, η^2^ = 0.06, Power = 0.65) and time (F(4,110) = 6.76, *p* < 0.001, η^2^ = 0.20; Power = 0.99) main effects were also significant. Post hoc tests showed that the exclusive breastfeeding group had higher scores than the formula group at 12 and 18 months of age. Regarding changes over time, the post hoc test showed that the formula group had declines in cognitive scores from 4 to 24 months and the mixed feeding group had declines from 4 to 12 and 24 months; no changes were observed for the exclusive breastfeeding group.

Regarding language development, a significant group by time interaction was found (Roy’s Largest Root = 0.11, F(4,111) = 2.96, *p* = 0.023, η^2^ = 0.10, Power = 0.77). The group (F(2,113) = 7.57, *p* < 0.001, η^2^ = 0.12, Power = 0.94) and time (F(4,110) = 16.77, *p* < 0.001, η^2^ = 0.38, Power = 1.00) main effects were also significant. Post hoc tests showed that the exclusive breastfeeding and mixed feeding groups had higher scores than the formula group at 12 months, and that at 18 months the exclusive breastfeeding group had higher scores than the formula group. Regarding changes over time, the post hoc test showed that the formula group had declines in language scores from 4 to 24 months, and the mixed feeding group had declines from 8 to 24 months; for the exclusive feeding group, declines were observed from 8 and 12 to 24 months.

**Figure 1:**
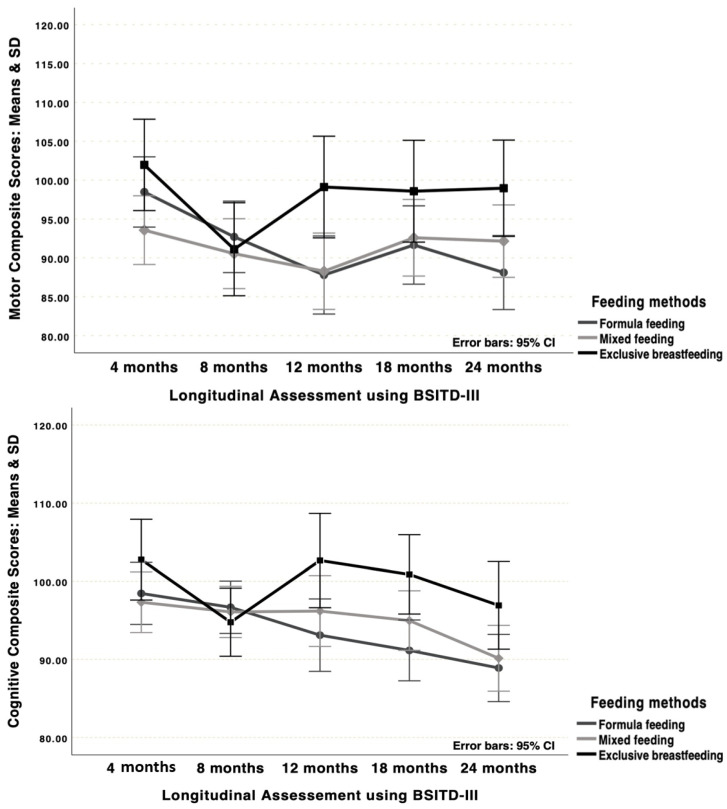

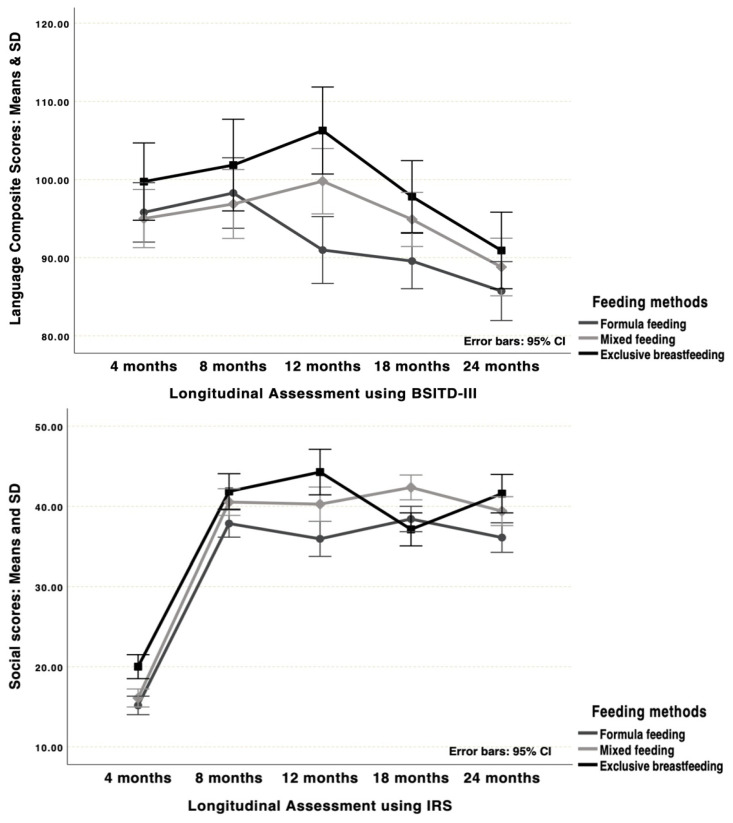
Child motor, cognitive, and language development (BSITD-III) and social development (IRS) over time by groups.

Regarding social development, a significant group by time interaction was detected (Roy’s Largest Root = 0.35, F(4,111) = 9.64, *p* < 0.001, η^2^ = 0.10, Power = 1.00). The group (F(2,113) = 20.47, *p* < 0.001, η^2^ = 0.27, Power = 1.00) and time (F(4,110) = 564.32, *p* < 0.001, η^2^ = 0.95, Power = 1.00) main effects were also significant. Post hoc tests showed that the exclusive breastfeeding group had higher scores than the formula group at 4, 8, 12, and 24 months, and that at 18 months mixed feeding had higher scores compared to the other two groups. Regarding changes over time, the post hoc test showed that the mixed feeding and formula groups showed increases in social development from 4 months to the subsequent ones—after 8 months, the scores remained stable. For the exclusive breastfeeding group, increases were observed from 4 months to the subsequent ones; decreases were observed from 12 to 18 months, with a new increase from 18 to 24 months. Figure 1 presents the child motor, cognitive, language, and social development trajectories by groups.

### 3.3. Maternal Parenting Skills and Mother/Child Dyads of Interactions: Groups Comparisons

Table 4 presents means and standard deviations as well as the post hoc tests for mothers’ parenting skills and mother/child dyads of interactions assessed using the IRS over time by groups.

Regarding maternal parenting skills, a significant group by time interaction was detected (Roy’s Largest Root = 0.13, F(4,111) = 3.49, *p* = 0.010, η^2^ = 0.11, Power = 0.85). The group (F(2,113) = 22.37, *p* < 0.001, η^2^ = 0.28, Power = 1.00) and time (F(4,110) = 195.55, *p* < 0.001; η^2^ = 0.88, Power = 1.00) main effects were significant. The post hoc tests showed that the exclusive breastfeeding group had higher scores than the formula group at 4, 8, 12, and 18 months and higher scores than the mixed feeding group at 4 months; the mixed feeding group had higher scores than the formula group at 4, 8, and 18 months. Regarding the time factor, the post hoc tests showed that for all groups the scores increased from 4 months to the subsequent ones.

Regarding mother/child dyads of interactions, a significant group by time interaction was detected (Roy’s Largest Root = 0.26, F(4,110) = 7.18, *p* < 0.001 η^2^ = 0.21, Power = 0.99). The group (F(2,112) = 29.32; *p* < 0.001; Power = 1.00) and time (F(4,109) = 152.40, *p* < 0.001; η^2^ = 0.84, Power = 1.00) main factors were significant. Post hoc tests showed that the exclusive breastfeeding group had higher scores than the formula group, and higher scores at 4 and 8 months than the mixed feeding group; the mixed feeding group had higher scores than the formula group at 4 and 12 months. Regarding the time factor, the post hoc tests showed that for all groups the scores increased from 4 months to the subsequent ones, although a decrease in score was observed for the exclusive breastfeeding group from 12 to 18 months, followed by a new subsequent increase to 24 months.

### 3.4. Longitudinal Associations Between Risk and Protective Factors and Developmental Outcomes

Longitudinal analyses revealed that various risk and protective factors were uniquely associated with developmental outcomes across time, highlighting the dynamic and evolving nature of these relationships throughout the developmental trajectory. To ensure the robustness and accuracy of the regression models used in the analysis, variables exhibiting multicollinearity beyond acceptable limits—specifically those with variance inflation factors (VIF) greater than 2.00—were excluded from the models. This step was taken to minimize redundancy among predictors and to enhance the interpretability and validity of the statistical findings.

Table 5 presents the results of multivariate regression analyses for the motor development outcome by groups. At 4 months, the variance in motor models was significantly explained differently across groups (exclusive breastfeeding, 18%; mixed feeding, 28%; formula, 24%) by environmental (father’s age; mother–child dyads; mother’s age and breastfeeding perception; siblings) and clinical (NICU stay) factors.

At 8 months, the variance in motor models was significantly explained differently across groups (exclusive breastfeeding, 10%; mixed feeding, 16%; formula, 6%) by environmental (family income; mother–child dyads) and clinical (leukomalacia; BPD) factors.

At 12 months, the variance in motor models was significantly explained differently across groups (exclusive breastfeeding, 20%; mixed feeding, 37%; formula, 26%) by environmental (mother–child dyads) and clinical (BPD; periventricular hemorrhage; mechanical ventilation) factors.

At 18 months, the variance in motor models was significantly explained differently across groups (exclusive breastfeeding, 20%; mixed feeding, 37%; formula, 26%) by environmental (mother–child dyads) and clinical (BPD; periventricular hemorrhage; mechanical ventilation) factors.

At 24 months, the variance in motor models was significantly explained differently across groups (exclusive breastfeeding, 20%; mixed feeding, 39%; formula, 27%) by environmental (mother–child dyads; breastfeeding length) and clinical (SGA; BPD; periventricular hemorrhage; mechanical ventilation; leukomalacia) factors.

Table 6 presents the results of multivariate regression analyses for the cognitive development outcome by groups. At 4 months, the variance in cognitive models was significantly explained differently across groups (exclusive breastfeeding, 24%; mixed feeding, 37%; formula, 17%) by environmental (father’s age and formal education; mother–child dyads; mother’s age and breastfeeding perception; siblings) and clinical (NICU stay) factors.

At 8 months, the variance in cognitive models was significantly explained differently across groups (exclusive breastfeeding, 26%; mixed feeding, 35%; formula, 13%) by environmental (father’s age and formal education; mother–child dyads; mother’s age, formal education, and breastfeeding perceptions; siblings) and clinical (BPD; NICU stay; periventricular hemorrhage) factors.

At 12 months, the variance in cognitive models was significantly explained differently across groups (exclusive breastfeeding, 49%; mixed feeding, 35%; formula, 26%) by environmental (mother’s age; mother–child dyads; siblings) and clinical (BPD; periventricular hemorrhage; mechanical ventilation; leukomalacia) factors.

At 18 months, the variance in cognitive models was significantly explained differently across groups (exclusive breastfeeding, 46%; mixed feeding, 44%; formula, 22%) by environmental (mother–child dyads; mother’s formal education) and clinical (BPD; periventricular hemorrhage; leukomalacia; NICU stay) factors.

At 24 months, the variance in cognitive models was non-significant for the exclusive breastfeeding group, with BPD explaining only 0.4% of the variance in the model. For the mixed feeding group and formula group, the models were significant and the variance (mixed feeding, 39%; formula, 34%) was explained by environmental (mother–child dyads; mother’s formal education and breastfeeding perceptions; siblings) and clinical (BPD; periventricular hemorrhage) factors.

Table 7 presents the results of multivariate regression analyses for the language development outcome by groups. At 4 months, the variance in language models was significantly explained differently across groups (exclusive breastfeeding, 41%; mixed feeding, 59%; formula, 18%) by environmental (father’s age and formal education; mother–child dyads; mother’s age and formal education; breastfeed length) and clinical (NICU stay; leukomalacia) factors.

At 8 months, the variance in language models was significantly explained differently across groups (exclusive breastfeeding, 11%; mixed feeding, 18%; formula, 13%) by environmental (family income; mother–child dyads; father’s age) and clinical (BPD) factors.

At 12 months, the variance in language development models was significantly explained differently across groups (exclusive breastfeeding, 23%; mixed feeding, 59%; formula, 14%) by environmental (mother’s age and formal education; mother–child dyads; breastfeeding length; father’s age and formal education) and clinical (BPD; NICU stay; leukomalacia) factors.

At 18 months, the variance in language development models was significantly explained differently across groups (exclusive breastfeeding, 33%; mixed feeding, 20%; formula, 22%) by environmental (mother–child dyads; father’s age; mother’s age; siblings) and clinical (NICU stay) factors.

At 24 months, the model was non-significant for the exclusive breastfeeding group, with siblings explaining only 2% of the variance in the model. For the mixed feeding group and formula group, the models were significant and the variance in the models (mixed feeding, 28%; formula, 31%) was explained by environmental (mother–child dyads; mother’s breastfeeding perceptions) and clinical (mechanical ventilation) factors. 

Table 8 presents the results of multivariate regression analyses for the social development outcome by groups. At 4 months, the variance in social models was significantly explained differently across groups (exclusive breastfeeding, 95%; mixed feeding, 84%; formula, 84%) by environmental (father’s age; mother–child dyads; siblings) and clinical (BPD) factors.

At 8 months, the variance in social models was significantly explained differently across groups (exclusive breastfeeding, 79%; mixed feeding, 77%; formula, 6%) by environmental (mother’s formal education; mother–child dyads; father’s age) and clinical (BPD) factors.

At 12 months, the variance in social development models was significantly explained differently across groups (exclusive breastfeeding, 87%; mixed feeding, 71%; formula, 76%) by environmental (mother–child dyads) and clinical (mechanical ventilation) factors.

At 18 months, the variance in social development models was significantly explained differently across groups (exclusive breastfeeding, 58%; mixed feeding, 86%; formula, 69%) by environmental (mother’s age; mother–child dyads; breastfeeding length) and clinical (BPD) factors.

At 24 months, the variance in motor development models was significantly explained differently across groups (exclusive breastfeeding, 45%; mixed feeding, 82%; formula, 80%) by environmental (family income; mother–child dyads; breastfeeding length) and clinical (BPD; NICU stay) factors.

## 4. Discussion

### 4.1. Group Characteristics: Neonatal Clinical and Environmental Factors

The initial data indicated similarities across groups in gestational and neonatal parameters—such as gestational age, birth weight, length, respiratory support, and head circumference—ensuring a comparable baseline and minimizing prenatal or perinatal bias. An exception was the difference in NICU stay, with exclusively breastfed infants spending less time in intensive care compared to those in the mixed and formula groups. This likely reflects better early health and fewer neonatal complications. However, since other perinatal parameters were similar across groups, the protective role of breastfeeding cannot be attributed solely to the initial clinical status. Rather, it reflects the interplay of biological vulnerabilities and environmental opportunities. Breastfeeding functions both as a biological buffer—providing nutrients essential for myelination and neural connectivity [12,13]—and as an environmental resource, fostering sensitive maternal responsiveness and high-quality dyadic interaction [6,12]. Thus, shorter NICU stays may mark not only better health but also earlier opportunities for caregiving practices that promote development. These findings reinforce the view that preterm development must be understood as a transactional process shaped by early clinical conditions and the caregiving environment [5,23].

Regarding environmental variables, groups showed similar characteristics in parental education, family income, prenatal visits, and obstetric history—factors known to influence child development. This suggests that families of preterm children across groups shared comparable backgrounds, and that breastfeeding practices were adopted relatively independently of socioeconomic status [22,23,24]. Maternal perceptions of breastfeeding experiences (MBFES, Table 3) were also comparable across groups, though mothers in the mixed group often introduced formula due to perceived nutritional demands. Prior studies indicate that exclusive breastfeeding is often experienced as more gratifying, given the stronger bond and perceived health benefits [6,20,22]. Nevertheless, maintaining breastfeeding—even with supplementation—appears to sustain maternal satisfaction, especially when adequate support is provided. These findings underscore the need for public policies that promote breastfeeding regardless of supplementation. Similarly, maternal knowledge of child development (KIDI) and caregiving practices (DAIS) were comparable across groups, suggesting that preterm mothers, in general, provide developmental opportunities in daily routines [19,21]. Although mothers in the exclusive breastfeeding group showed slightly higher averages, the lack of statistical significance suggests a trend that warrants further study. Finally, the AHEMD results showed only one difference: families in the mixed group provided more fine motor toys. Other domains, including physical space and stimulus variety, did not differ across groups, indicating that, while feeding practices may influence certain aspects of the home environment, they do not necessarily affect overall developmental opportunities.

### 4.2. Child Development, Mother Parenting Skills, and Mother–Child Interactions over Time

*Motor Development.* The analyses revealed a non-significant group-by-time interaction for motor development, but both group and time main effects were significant. Post hoc comparisons showed that the exclusive breastfeeding group performed better than the mixed and formula groups at 12 months, and better than the formula group at 24 months. These results suggest that exclusive breastfeeding confers early motor advantages, consistent with evidence linking human milk to neurodevelopment [12,16]. Breast milk contains bioactive components such as long-chain polyunsaturated fatty acids and sphingomyelin, which are essential for the myelination of motor pathways [13,15], potentially explaining the higher performance observed in the second year of life.

The absence of sustained differences across all time points highlights the growing importance of environmental opportunities for exploration and play beyond infancy [5,6]. The decline in motor performance in the formula group may reflect the combined effects of nutritional disadvantage and reduced relational stimulation during feeding [11,12]. Overall, these findings reinforce a bioecological perspective in which motor development emerges from the interplay of biological vulnerabilities and contextual resources, with breastfeeding providing an early protective buffer that must be complemented by enriched caregiving to sustain progress [23].

*Cognitive Development.* The analyses revealed a significant group-by-time interaction for cognitive development, showing that feeding type influenced developmental trajectories across time. Post hoc comparisons indicated that the exclusive breastfeeding group consistently outperformed the formula group at 12, 18, and 24 months, with the mixed feeding group showing intermediate results. These findings emphasize the protective role of exclusive breastfeeding, which supported stable cognitive outcomes, while both mixed and formula groups exhibited declines.

This advantage aligns with prior evidence linking breast milk to improved neurodevelopment in preterm children [17,25]. Breast milk provides nutrients critical for myelination and neural connectivity (Dinleyici et al., 2023; Peila, Riboldi, and Coscia, 2024), while breastfeeding interactions supply rich cognitive stimulation through sensitive caregiver–infant exchanges [6,11,12]. The declines observed in formula-fed and mixed groups suggest that the absence of exclusive breastfeeding may deprive children of both biological and relational inputs essential for cognitive growth. Taken together, these findings highlight exclusive breastfeeding as an important early buffer against biological risks, supporting long-term cognitive trajectories in preterm children.

*Language Development.* The analyses revealed a significant group-by-time interaction for language development, indicating differential trajectories by feeding type. Post hoc tests showed that exclusive and mixed breastfeeding groups performed better than the formula group at 12 months, with exclusive breastfeeding maintaining an advantage at 18 months. These results suggest that breast milk—exclusive or partial—supports early language acquisition, particularly during the second year of life.

The protective influence of breastfeeding is consistent with evidence that breast milk provides nutrients essential for myelination and neural networks underpinning speech and communication [12,13]. Beyond nutrition, breastfeeding fosters communicative exchanges, maternal responsiveness, and vocal interactions that stimulate early receptive and expressive skills [6,12]. Despite these benefits, language scores declined after the first year in all groups, most markedly in the formula group, moderately in the mixed group, and mildly in the exclusive breastfeeding group. By 24 months, the diminishing group differences suggest that parental education, quality of stimulation, and sibling interactions become increasingly important [5,7]. Thus, breastfeeding provides an early advantage for language development, but sustained environmental support is needed to consolidate and extend these gains.

*Social Development.* The analyses revealed a significant group-by-time interaction for social development, showing that feeding type shaped early social trajectories. At most time points, the exclusive breastfeeding group outperformed the formula group, with mixed feeding showing a temporary advantage at 18 months, when exclusive breastfeeding scores dipped before recovering at 24 months.

These findings highlight the role of breastfeeding and dyadic interactions in fostering early social development. Breastfeeding promotes frequent emotional and physical exchanges, which enhance attachment, security, and responsiveness—key foundations for later social competence [6]. Beyond nutrition, breastfeeding creates relational contexts that coordinate motor and cognitive behaviors relevant for social engagement [5,23].

The temporary decline at 18 months likely reflects the non-linear nature of social development, marked by periods of consolidation and reorganization. This stage often coincides with weaning and greater emotional challenges, including separation anxiety, which may temporarily affect social skills. The recovery observed at 24 months suggests that this decline is transitional rather than regressive, consistent with developmental frameworks that emphasize discontinuity and context dependence [7]. Overall, the results underline the sensitivity of social development to caregiving quality, with breastfeeding creating favorable conditions for relational security and competence while underscoring the need for sustained caregiver support during critical transitions.

*Maternal Parenting Skills.* Regarding maternal parenting skills, a significant group-by-time interaction was found, along with significant group differences, with the exclusive breastfeeding group showing higher scores in four of the five time points. Across time, all groups improved, particularly in the early months, with stability from 12 to 24 months. These findings suggest that mothers who exclusively breastfed their children exhibited more effective parenting skills, especially in perceiving and responding to their children’s needs and promoting autonomy, consistent with previous studies [6,20,22]. This pattern supports the idea that maternal skills are enhanced by the close physical and emotional contact provided during breastfeeding, which facilitates sensitive and responsive caregiving [6,12,23].

*Mother–Child Dyadic Interactions.* For dyadic interactions, significant group-by-time interactions were also detected. Group differences were evident, with the exclusive breastfeeding group showing higher scores in four of five assessments—at 4, 8, 12, and 18 months. The time factor was significant, with scores increasing from 4 to 8 months, followed by stability across groups. The temporary decline observed in the exclusive breastfeeding group from 12 to 18 months, followed by recovery at 24 months, suggests a transitional phase in interaction quality, possibly reflecting the challenges of weaning and developmental reorganization. Dyadic interactions—assessed by the quality of exchanges between mothers and children—were more effective in the exclusive breastfeeding group, underscoring breastfeeding’s role in fostering emotional bonding and reciprocal interactions. The physical and emotional closeness facilitated by breastfeeding may create opportunities for richer, more responsive interactions with long-term effects on relationship quality [19,21]. These results reinforce the critical role of breastfeeding not only in nutritional support but also in strengthening parenting skills and dyadic interactions—protective processes that may enhance social and emotional outcomes in preterm children [7,17].

*Integrative Approach to Child–Mother Changes Over Time.* Taken together, the findings across developmental domains highlight the dynamic and transactional nature of preterm children’s development, in which early biological vulnerabilities and environmental opportunities constantly interact. Across motor, cognitive, language, and social trajectories, exclusive breastfeeding consistently emerged as an early protective factor, buffering clinical risks such as prolonged NICU stays, bronchopulmonary dysplasia, and neurological complications, while also fostering opportunities for enriched stimulation through close caregiver–child contact. Yet, declines observed in language and temporary dips in social development emphasize that breastfeeding alone is not sufficient; sustained environmental opportunities, including parental education, family resources, and sibling interactions, are critical for maintaining developmental progress. Importantly, maternal parenting skills and the quality of mother–child dyadic interactions mirrored these child outcomes, showing higher scores among exclusively breastfeeding dyads and evolving over time toward greater responsiveness and reciprocity. This parallel trajectory reinforces the bioecological view that children’s development and caregiving practices are interdependent, with breastfeeding functioning not only as nutrition but also as a relational context that nurtures emotional security, social competence, and cognitive stimulation [6,12]. Altogether, these results underscore the importance of integrated strategies that address clinical vulnerabilities while promoting responsive caregiving, thereby maximizing developmental potential in preterm children across the first two years of life.

### 4.3. Associations Between Risks and Protective Factors: Motor, Cognitive, Language, and Social Development of Preterm Children

*Risks and Protective Factors and Motor Development Outcome*. The regression analyses highlight how biological and environmental factors interact in shaping motor development in preterm children, with their influence shifting across the first two years of life. In early infancy, breastfeeding and the quality of mother–child dyadic interactions emerged as protective factors, supporting the establishment of basic sensorimotor functions and providing both nutritional and relational benefits [5,20]. Beyond immunological protection, breast milk contributes nutrients essential for myelination and neural connectivity in motor pathways, such as the corticospinal tract and cerebellum [12,15]. These findings reinforce evidence that breastfeeding can partly compensate for early vulnerabilities, especially when mothers perceive breastfeeding positively and engage responsively [12,19]. Additional protective contributions stemmed from siblings, who provide opportunities for social play and motor exploration [20], and from socioeconomic resources such as family income [4].

At the same time, clinical risks were evident early on, with prolonged NICU stays associated with poorer motor outcomes [4,7]. As children approached 8–12 months, when motor demands became more complex with antigravity postures and locomotion, neonatal morbidities—including bronchopulmonary dysplasia, small for gestational age (SGA), leukomalacia, periventricular hemorrhage, and prolonged mechanical ventilation—exerted progressively stronger negative effects. These results echo the literature showing that prematurity and low birth weight increase the risk of delayed motor milestones and long-term impairments [2,3]. Importantly, these effects are most detrimental when infants must master advanced tasks requiring coordination, balance, and strength.

*Risks and Protective Factors and Cognitive Development Outcome.* The regression results emphasize the crucial role of both clinical and environmental factors across the first two years of life for cognitive development. In early infancy, prolonged NICU stays were associated with poorer outcomes, while conditions such as bronchopulmonary dysplasia and periventricular hemorrhage contributed to growing vulnerabilities over time. These findings are consistent with evidence showing that prematurity and low birth weight increase the likelihood of developmental impairments due to organ immaturity [1,2,3]. The persistence of clinical risks underscores the importance of long-term follow-up, as emphasized by studies linking neonatal morbidities to later cognitive and behavioral challenges [2,7].

In parallel, environmental resources—including parental education, the father’s age, maternal perceptions of breastfeeding, sibling presence, and especially mother–child dyadic interactions—acted as protective factors. These findings converge with prior evidence showing that parental education, family income, and the quality of the relational environment significantly shape cognitive outcomes [4,5,6]. Mother–child dyadic interactions consistently predicted better outcomes, echoing evidence that maternal sensitivity and responsiveness are especially important for preterm children [8,12].

Exclusive breastfeeding in the first year enhanced cognitive trajectories, especially when combined with positive maternal perceptions and enriched caregiving contexts. This aligns with studies showing that breast milk provides nutrients essential for myelination and neural connectivity, as well as opportunities for high-quality interactions that can partly offset biological risks [12,13,17,19].

*Risks and Protective Factors and Language Development Outcome.* The regression analyses indicate that language development in preterm children is shaped by the interplay of biological, environmental, and social factors, with their relative contributions shifting over time. In the first months, breastfeeding and maternal parenting skills provided not only nutrition but also crucial emotional and communicative stimuli for early language acquisition. This supports evidence that breastfeeding enhances neurodevelopment beyond nutrition, strengthening neural connectivity for communication and language processing [11,12]. Early predictors in our models—such as parental education, maternal perceptions of breastfeeding, and dyadic interactions—converge with prior studies showing that sensitive caregiving and responsive exchanges build a foundation for linguistic growth [6,12].

From 8 to 12 months, family income, parental education, and dyadic interactions supported word recognition and communicative gestures, while clinical adversities such as BPD, NICU stays, and leukomalacia exerted negative influences. This resonates with evidence that prematurity and neonatal complications are strongly associated with expressive and receptive delays [17,25]. From 18 months onward, siblings, the father’s age, and mother–child interactions contributed to language growth. By 24 months, exclusive breastfeeding no longer predicted outcomes, showing that, while it confers early advantages, sustained stimulation is essential for consolidating skills.

*Risks and Protective Factors and Social Development Outcome.* The regression analyses demonstrated that social development is explained by a combination of environmental and clinical factors, with breastfeeding, parental resources, and dyadic interactions emerging as consistent predictors. In early infancy, exclusive breastfeeding and close mother–child interactions provided a relational foundation for social engagement, while siblings and paternal characteristics added opportunities for play. These findings align with research showing that breastfeeding fosters maternal sensitivity, responsiveness, and emotional availability—qualities critical for socioemotional growth [6,12,19].

From 8 to 12 months, maternal education and dyadic exchanges supported gestures, joint attention, and reciprocal exchanges, while clinical risks such as BPD and mechanical ventilation negatively influenced outcomes [2,7]. Despite these risks, enriched family environments buffered adverse effects, echoing findings that parental education, family income, and caregiving quality mitigate clinical vulnerabilities [4,5]. By 18–24 months, children’s emerging social skills began to exert a stronger influence, marking a shift from reliance on parental stimuli toward greater independence in exploration and problem-solving. This suggests that social development acts as a bridge domain, linking motor and cognitive growth through engagement with the environment [5,7].

*Integrative Approach to Risks and Protective Factors and Neurodevelopment Interactions Across Domains Over Time.* Across domains, the associations reveal a consistent pattern: early clinical adversities—such as prolonged NICU stay, BPD, SGA, leukomalacia, and hemorrhage—impose significant risks, but protective influences including exclusive breastfeeding, parental education, sibling interactions, and especially responsive mother–child dyads repeatedly emerged as buffers. Breastfeeding operated as both a biological and relational factor, supplying essential nutrients while fostering sensitive caregiving and emotional availability. Over time, environmental and social opportunities—such as parental education, family income, and sibling interactions—became increasingly important as developmental demands grew more complex. This bioecological interplay underscores that preterm development is shaped not by biology or caregiving alone, but by their continuous interaction. Interventions must therefore integrate medical follow-up with strategies that enhance responsive caregiving, parental education, and enriched environments to mitigate risks and maximize potential across all domains.

## 5. Strengths and Limitations

This study has several strengths. It is one of the few longitudinal investigations in a middle-income context exploring how breastfeeding and mother–child interactions jointly influence multiple domains of neurodevelopment in preterm children during the first two years of life. The study design, with repeated measures across five time points, enable a detailed understanding of their differential effects on developmental trajectories. Importantly, this research examined three distinct feeding practices (exclusive breastfeeding, mixed feeding, and formula feeding), providing a distinct understanding of their differential effects—an area that remains underexplored in the literature. Furthermore, the study uniquely integrated the role of mother–child interactions as both a relational protective factor and a mechanism shaping developmental outcomes. By addressing a population of preterm children facing extreme biological and social vulnerabilities, this work provides rare and valuable evidence of how caregiving and feeding practices buffer risk. The use of validated instruments and rigorous statistical analyses further supports the robustness of the findings.

Nonetheless, some limitations should be acknowledged. The sample size, although sufficient to detect group differences, was relatively modest and recruited from a single center, which may limit generalizability. Also, because this study was carried out in a follow-up clinic exclusively dedicated to preterm infants, the establishment of a full-term control group was not feasible. Additionally, the non-significant differences in mean GA and BW between the three groups could have influenced the outcomes. In addition, outcomes beyond 24 months were not assessed, preventing conclusions about longer-term effects of early feeding practices. Future research should replicate these findings in larger and more diverse populations, extend follow-up into school age, and test the effectiveness of targeted interventions designed to enhance breastfeeding support and sensitive caregiving in vulnerable families.

## 6. Conclusions

This study demonstrated that protective factors such as exclusive breastfeeding, responsive mother–child interactions, and family resources play a decisive role in the motor, cognitive, language, and social development of preterm children. A novel contribution of this work is the evidence, from a longitudinal study in a low- to middle-income setting, that exclusive breastfeeding during the first two years of life was associated with better developmental outcomes than formula feeding, with mixed feeding showing intermediate results. Future research should extend follow-up beyond early childhood and evaluate interventions that combine breastfeeding support with programs fostering sensitive caregiving. Taken together, these findings advance the understanding of the mechanisms that shape preterm development and reinforce the importance of public policies that promote breastfeeding, parental education, and responsive caregiving as strategies to mitigate risks, enhance skills, and improve the quality of life of this vulnerable population.

## Figures and Tables

**Table 1:** Premature children’s clinical factors: group comparison over time.

Clinical Factors	Premature Children Groups (N = 116)			Group Comparisons *p*
	Exclusive Breastfeeding (N = 26)	Mixed Feeding (N = 46)	Formula (N = 44)	
	M (SD)			
Gestational age (weeks)	31.05 (3.64)	29.67 (2.84)	29.87 (3.27)	0.086
Weight at birth (grams)	1458.46 (778.12)	1301.47 (754.18)	1295.65 (560.82)	0.561
Length at birth (cm)	39.61 (4.58)	37.78 (4.71)	38.43 (4.70)	0.114
Cephalic perimeter at birth (cm)	27.56 (2.90)	26.65 (3.05)	27.03 (2.70)	0.221
APGAR 5th minute	8.19 (1.13)	7.43 (1.82)	7.41 (1.94)	0.145
NICU stay (days)	44.85 (25.90)	62.87 (29.45)	64.59 (32.78)	**0.025**
Mechanical ventilation (days)	1.15 (4.80)	5.63 (11.35)	7.52 (16.16)	0.135
CPAP (days)	2.11 (4.72)	3.89 (6.21)	2.84 (4.78)	0.414
Parenteral nutrition	5.64 (5.42)	12.41 (12.51)	12.59 (16.62)	0.077
	N (%)			
Sex Boys	11 (42.3)	25 (54.3)	21 (45.7)	0.600
Girls	15 (57.7)	21 (45.7)	23 (52.3)	
Small for gestational age	6 (23.1)	10 (21.7)	10 (22.7)	0.472
Bronchopulmonary dysplasia	3 (11.5)	11 (23.9)	13 (29.5)	0.225
Late sepsis	6 (23.1)	19 (41.3)	16 (36.4)	0.294
Periventricular hemorrhage				
Grade I	3 (11.5)	9 (19.6)	9 (20.5)	0.710
Grade II	2 (7.7)	3 (6.5)	4 (9.1)	
Grade III	--	2 (4.3)	2 (4.5)	
Grade IV	1 (3.8)	3 (6.5)	--	
Leukomalacia	1 (3.8)	4 (8.7)	4 (9.1)	0.851

Note. NICU: Neonatal Intensive Care Unit; CPAP: continuous positive airway pressure; N: number of participants; M: mean; SD: standard deviation; significant results are presented in bold.

**Table 2:** Premature children environmental and familiar outcomes: group comparison over time.

Environmental and Familiar Factors	Premature Children Groups (N = 116)			Group Comparisons *p*
	Exclusive Breastfeeding (N = 26)	Mixed Feeding (N = 46)	Formula (N = 44)	
	M (SD)			
Breastfeeding length (months)	13.65 (6.40)	11.52 (10.35)	--	0.345
Mother’s age at birth (years)	30.08 (6.25)	29.45 (5.52)	29.52 (6.89)	0.931
Father’s age at birth (years)	32.40 (6.66)	32.09 (6.49)	31.96 (6.75)	0.973
Family income (BRL)	2756.25 (729.35)	2690.90 (1894.95)	2678.12 (2026.55)	0.978
Number of previous gestations	1.23 (1.45)	1.56 (142)	1.48 (1.63)	0.751
Number of prenatal visits (n)	5.27 (2.66)	5.59 (2.33)	5.34 (2.37)	0.600
MBFES—Breastfeeding experience	105.42 (11.43)	100.57 (11.29)	--	0.082
DAIS—Maternal practices	14.81 (9.08)	9.76 (8.67)	11.93 (7.61)	0.052
KIDI—Parental knowledge	0.51 (0.26)	0.38 (0.29)	0.47 (0.26)	0.112
AHEMD-IS opportunities				
Home physical space	2.58 (1.60)	2.13 (1.50)	2.64 (1.57)	0.260
Stimuli variety	13.78 (1.55)	14.31 (1.21)	13.92 (1.46)	0.216
Fine motor toys	10.92 (6.60)	16.65 (8.76)	11.20 (6.20)	**0.001**
Gross motor toys	8.00 (1.91)	9.13 (2.72)	8.16 (2.83)	0.117
Total score	30.53 (9.08)	32.15 (10.82)	28.66 (9.73)	0.574
	N (%)			
Mother’s formal education				
Primary school	--	3 (8.1)	5 (14.3)	0.293
Elementary school	3 (20.0)	9 (24.3)	10 (28.6)	
High school	10 (66.6)	24 (64.9)	15 (42.8)	
Higher education	2 (13.3)	1 (2.7)	5 (14.3)	
Father’s formal education				
Primary school	2 (13.3)	4 (11.4)	2 (6.5)	0.505
Elementary school	4 (26.7)	12 (34.3)	11 (35.5)	
High school	9 (60.0)	14 (40.0)	12 (38.7)	
Higher education	--	5 (14.3)	6 (19.3)	
Previous premature births	3 (11.5)	2 (4.3)	2 (4.5)	0.491
Type of delivery				
Natural	8 (30.8)	15 (32.6)	16 (36.4)	0.876
Cesarean	18 (69.2)	31 (67.4)	28 (63.6)	
Preeclampsia (yes)	8 (30.8)	17 (37.0)	11 (25.0)	0.472

Note. MBFES: Maternal Breastfeeding Evaluation Scale; DAIS: Daily Activities of Children Scale; KIDI: Knowledge of Children Development Inventory; AHEMD-IS: Affordances in the Home Environment for Motor Development–Children Scale; N: number of participants; M: mean; SD: standard deviation; significant results are presented in bold.

**Table 3:** Child motor, cognitive, language, and social development: group comparisons over time.

	M (SD) Premature Children Groups (N = 116)			Group Factor Post Hoc Tests *p*
BSITD-III	Exclusive Breastfeeding (N = 26)	Mixed Feeding (N = 46)	Formula (N = 44)	
Motor Scores				
4 months (time 1)	101.96 (11.44)	93.56 (14.58)	98.48 (17.40) ^t1#3 and 5^	0.067
8 months (time 2)	91.11 (12.49)	90.54 (13.74)	92.70 (18.29)	0.794
12 months (time 3)	99.11 (9.97) ^gA#B and C^	88.28 (16.26) ^gB^	87.79 (20.22) ^gC,t3^	**0.015**
18 months (time 4)	98.58 (14.98)	92.58 (15.62)	91.66 (19.01)	0.226
24 months (time 5)	98.96 (11.11) ^gA#C^	92.15 (16.73)	88.11 (17.40) ^gC,t5^	**0.026**
**Time Factor Post Hoc Tests *p***	0.100	0.191	**0.002**	
Cognitive Scores				
4 months (time 1)	102.77 (10.25)	97.33 (10.60) ^t1#5^	98.45 (16.88) ^t1#4,5^	0.239
8 months (time 2)	94.77 (9.13)	95.09 (10.32)	96.68 (13.06) ^t2#5^	0.788
12 months (time 3)	102.65 (10.99) ^gA#C^	96.19 (14.91) ^t3#5^	93.11 (18.16) ^gC^	**0.049**
18 months (time 4)	100.88 (15.55) ^gA#C^	94.98 (11.79)	91.16 (12.70) ^t4,gC^	**0.013**
24 months (time 5)	96.92 (6.34)	90.15 (15.03) ^t5^	88.90 (16.93) ^t5^	0.070
**Time Factor Post Hoc Tests *p***	0.250	**0.021**	**0.002**	
Language Scores				
4 months (time 1)	99.73 (10.35)	95.00 (12.64)	95.79 (14.00) ^t1#5^	0.300
8 months (time 2)	101.85 (14.95) ^t2#5^	96.87 (15.23) ^t2#5^	98.27 (15.04) ^t2#4,5^	0.405
12 months (time 3)	106.27 (18.04) ^gA#C;t3#5^	99.78 (11.60) ^gB#C;t3#5^	90.98 (14.45) ^gC^	**<0.001**
18 months (time 4)	97.81 (16.38) ^gA#C^	94.89 (11.50) ^t4#5^	89.57 (8.64) ^t4,gC^	**0.014**
24 months (time 5)	90.92 (6.01) ^t5^	88.80 (13.02) ^t5^	85.72 (14.81) ^t5^	0.227
**Time Factor Post Hoc Tests *p***	**<0.001**	**<0.001**	**<0.001**	
Social Scores				
4 months (time 1)	20.00 (2.19) ^gA#B,C^	16.09 (3.14) ^gB^	15.16(5.08) ^gC,t1#2,3,4,5^	**<0.001**
8 months (time 2)	41.85 (2.33) ^gA#C^	40.52 (6.28)	37.84 (6.40) ^gC^	**0.012**
12 months (time 3)	44.27 (2.55) ^gA#C^	40.26 (7.76) ^gB#C^	35.93 (8.62) ^gC^	**<0.001**
18 months (time 4)	37.11 (6.32 )^gA#B^	42.35 (5.75) ^gB#C^	38.41 (4.02) ^gC^	**<0.001**
24 months (time 5)	41.58 (3.02) ^gA#C^	39.39 (6.48) ^gB#C^	36.09 (7.14) ^gC^	**0.001**
**Time Factor Post Hoc Test *p***	**<0.001**	**<0.001**	**<0.001**	

Note. t represents the time comparisons (time 1 = 4 months; time 2 = 8 months; time 3 = 12 months; time 4 = 18 months; time 5 = 24 months); g represents the group comparisons (A: exclusive breastfeeding; B: mixed feeding; C: formula feeding); N: number of participants; M: mean; SD: standard deviation; # groups’ differences; significant results are presented in bold.

**Table 4:** IRS—Maternal parental skills and mother/child dyads of interactions: group comparison over time.

	M (SD) Premature Children Groups (N = 116)			Group Factor Post Hoc Test *p*
	Exclusive Breastfeeding (N = 26)	Mixed Feeding (N = 46)	Formula (N = 44)	
Mother’s Social Skills				
4 months (time 1)	40.65 (3.17) ^gA#B,C, t1#2,3,4,5^	35.98 (6.10) ^gB#C, t1#2,3,4,5^	29.27 (10.22) ^gC,t1#2,3,4,5^	**<0.001**
8 months (time 2)	62.92 (4.24) ^gA#C, t2^	59.26 (9.59) ^gB#C,t2^	53.95 (11.02) ^gC,t2^	**<0.001**
12 months (time 3)	61.00 (4.37) ^gA#C,t3^	56.65 (11.23) ^t3^	53.89 (12.88) ^gC,t3^	**0.033**
18 months (time 4)	61.46 (5.87) ^gA#C,t4^	60.89 (7.22) ^gB#C,t4^	55.59 (7.15) ^gC,t4^	**<0.001**
24 months (time 5)	58.50 (6.07) ^t5^	60.02 (10.22) ^t5^	56.98 (10.22) ^t5^	0.316
**Time Factor Post Hoc Test (*p*)**	**<0.001**	**<0.001**	**<0.001**	
Mother/Child Dyads				
4 months (time 1)	109.30 (8.54) ^gA#B,C, t1#2,3,4,5^	93.31 (14.70) ^gB#C,t1#2,3,4,5^	80.91 (24.06) ^gC, t1#2,3,4,5^	**<0.001**
8 months (time 2)	159.46 (8.23) ^gA#B,C,t2#t4^	144.60 (20.46) ^gB,t2^	134.98 (23.35) ^gC,t2^	**<0.001**
12 months (time 3)	153.54 (9.82) ^gA#C,t3^	141.38 (23.08) ^t3^	132.07 (27.99) ^gC,t3^	**<0.001**
18 months (time 4)	144.35 (16.30) ^t4^	151.69 (17.50) ^gB#C,t4^	137.66 (14.05) ^gC,t4^	**<0.001**
24 months (time 5)	145.04 (10.27) ^t5^	145.34 (21.78) ^t5^	136.64 (22.27) ^t5^	0.086
**Time Factor Post Hoc Test (*p*)**	**<0.001**	**<0.001**	**<0.001**	

Note. t represents the time comparisons across ages (time 1 = 4 months; time 2 = 8 months; time 3 = 12 months; time 4 = 18 months; time 5 = 24 months); g: group comparisons (A: exclusive breastfeeding; B: mixed feeding; C: formula feeding); N: number of participants; M: mean; SD: standard deviation; # groups’ differences; significant results are presented in bold.

**Table 5:** Multivariate linear regression by groups: motor development outcome.

Motor	Linear Regression Analysis: Formula Group/Longitudinal by Months		Unstandardized B	Standardized B	*p*	Partial Correlation
**Exclusive breastfeeding**	4-month model: R = 0.46, Adjusted R^2^ = 0.18, Durbin Watson = 1.50, ANOVA F(1,24) = 6.61, *p* = **0.017**					
	Predictor	Father’s age	1.05	0.46	**0.017**	0.46
	8-month model: R = 0.37, Adjusted R^2^ = 0.10, Durbin Watson = 1.73, ANOVA F(1,24) = 3.92, *p* = **0.050**					
	Predictor	Family income	−0.008	0.37	**0.050**	0.37
	12-month model: R = 0.48, Adjusted R^2^ = 0.20, Durbin Watson = 2.09, ANOVA F(1,24) = 7.35, *p* = **0.012**					
	Predictor	BPD ^yes^	−14.83	−0.48	**0.012**	−0.48
	18-month model: R = 0.56, Adjusted R^2^ = 0.29, Durbin Watson = 1.92, ANOVA F(1,24) = 11.26, *p* = **0.003**					
	Predictor	Mother–Child dyads	0.52	0.56	**0.003**	0.56
	24-month model: R = 0.52, Adjusted R^2^ = 0.20, Durbin Watson = 1.92, ANOVA F(2,23) = 4.20, *p* = **0.028**					
	Predictors	Mother–Child dyads	0.51	0.47	**0.016**	0.47
		SGA^yes^	−8.65	−0.33	0.080	−0.36
**Mixed** **feeding**	4-month model: R = 0.59, Adjusted R^2^ = 0.28, Durbin Watson = 1.72, ANOVA F(4,41) = 5.44, *p* < **0.001**					
	Predictors	Siblings ^number^	4.05	0.39	**0.008**	0.40
		Mothers breastfeeding perceptions	0.50	0.398	**0.004**	0.43
		Mother’s age	−1.01	−0.37	**0.011**	−0.38
		Mother–Child dyads	0.22	0.22	0.097	0.26
	8-month model: R = 0.44, Adjusted R^2^ = 0.16, Durbin Watson = 2.00, ANOVA F(2,43) = 5.30, *p* = **0.009**					
	Predictors	Leukomalacia ^yes^	−15.99	−0.33	**0.020**	−0.34
		Mother–Child dyads	0.17	0.26	0.069	0.27
	12-month model: R = 0.63, Adjusted R^2^ = 0.37 Durbin Watson = 1.67, ANOVA F(2,42) = 13.96, *p* < **0.001**					
	Predictors	Periventricular hemorrhage ^I–IV^	−6.14	−0.45	**0.001**	−0.47
		Mother–Child dyads	0.22	0.31	**0.022**	0.34
	18-month model: R = 0.68, Adjusted R^2^ = 0.41, Durbin Watson = 2.44, ANOVA F(4,41) = 8.74, *p* < **0.001**					
	Predictors	Mother’s formal education	2.15	0.35	**0.007**	0.40
		NICU stay ^yes^	−0.18	−0.33	**0.012**	−0.38
		Periventricular hemorrhage ^I–IV^	−3.43	−0.26	**0.048**	−0.30
		DBP ^yes^	−11.93	−0.22	0.088	−0.26
	24-month model: R = 0.67, Adjusted R^2^ = 0.39, Durbin Watson = 1.69, ANOVA F(4,41) = 8.24, *p* < **0.001**					
	Predictors	Periventricular hemorrhage ^I–IV^	−4.78	−0.34	**0.012**	−0.38
		Mechanical ventilation ^days^	−0.47	−0.32	**0.015**	−0.37
		Mother–Child dyads	0.20	0.26	**0.031**	0.33
		Breastfeeding length	0.37	0.24	**0.047**	0.30
**Formula**	4-month model: R = 0.57, Adjusted R^2^ = 0.24, Durbin Watson = 1.87, ANOVA F(5,38) = 3.73, *p* = **0.008**					
	Predictors	Mother’s age	−2.25	−0.85	**<0.001**	−0.53
		Father’s age	2.04	0.69	**0.001**	0.49
		Siblings ^number^	3.33	0.31	**0.050**	0.31
		Mother–Child dyads	0.19	0.26	0.069	0.29
		NICU stay ^days^	−0.13	−0.24	0.092	−0.27
	8-month model: R = 0.29, Adjusted R^2^ = 0.06, Durbin Watson = 2.07, ANOVA F(1,42) = 3.93, *p* = **0.050**					
	Predictor	BPD ^yes^	−11.59	−0.29	**0.050**	−0.29
	12-month model: R = 0.54, Adjusted R^2^ = 0.26, Durbin Watson = 1.51, ANOVA F(2,41) = 8.62, *p* < **0.001**					
	Predictors	Mechanical ventilation ^days^	−0.56	−0.45	**0.001**	−0.47
		BPD ^yes^	−20.93	−0.30	**0.027**	−0.34
	18-month model: R = 0.37, Adjusted R^2^ = 0.09, Durbin Watson = 1.83, ANOVA F(2,41) = 3.22, *p* = **0.050**					
	Predictors	Mother–Child dyads	0.40	0.29	**0.050**	0.30
		Periventricular hemorrhage ^I–IV^	−5.29	−0.24	0.112	−0.25
	24-month model: R = 0.56, Adjusted R^2^ = 0.27, Durbin Watson = 1.97, ANOVA F(3,40) = 6.25, *p* = **0.001**					
	Predictors	Leukomalacia ^yes^	−21.57	−0.36	**0.009**	−0.40
		Mother–Child dyads	0.25	0.32	**0.021**	0.36
		Periventricular hemorrhage ^I–IV^	−5.21	−0.25	0.058	−0.29

Note. Units of measurement: “Yes” indicates children with bronchopulmonary dysplasia (BPD); I–IV indicate periventricular hemorrhage grades; number indicates number of siblings at home; and days indicates days on mechanical ventilation. Significant results are presented in bold.

**Table 6:** Multivariate linear regression by groups: cognitive development outcome.

Cognitive	Linear Regression Analysis: Formula Group/Longitudinal by Months		Unstandardized B	Standardized B	*p*	Partial Correlation
**Exclusive breastfeeding**	4-month model: R = 0.52, Adjusted R^2^ = 0.24, Durbin Watson = 1.50, ANOVA F(1,24) = 8.82, *p* = **0.007**					
	Predictor	Father’s age	1.05	0.52	**<0.001**	0.52
	8-month model: R = 0.59, Adjusted R^2^ = 0.26, Durbin Watson = 2.05, ANOVA F(3,22) = 3.95, *p* = **0.021**					
	Predictors	Mother’s formal education	1.68	0.39	**0.044**	0.37
		Father’s age	0.67	0.37	**0.049**	0.36
		BPD ^yes^	−9.27	−0.33	0.072	−0.32
	12-month model: R = 0.74, Adjusted R^2^ = 0.49, Durbin Watson = 2.09, ANOVA F(3,22) = 9.08, *p* < **0.001**					
	Predictors	BPD ^yes^	−19.38	−0.57	**<0.001**	−0.56
		Mother’s age	0.77	0.41	**0.009**	0.41
		Mother–Child dyads	0.28	0.25	1.00	0.24
	18-month model: R = 0.71, Adjusted R^2^ = 0.46, Durbin Watson = 2.20, ANOVA F(2,23) = 11.50, *p* < **0.001**					
	Predictors	Mother–Child dyads	0.46	0.48	**0.004**	0.47
		BPD ^yes^	−18.86	−0.39	**0.017**	−0.38
	24-month model: R = 0.21, Adjusted R^2^ = 0.004, Durbin Watson = --, ANOVA F(1,24) = 1.09, *p* = 0.306					
	Predictor	DBP ^yes^	−4.06	−0.21	0.306	−0.21
**Mixed feeding**	4-month model: R = 0.67, Adjusted R^2^ = 0.37, Durbin Watson = 2.01, ANOVA F(6,39) = 5.34, *p* < **0.001**					
	Predictors	Siblings ^number^	3.06	0.41	**0.004**	0.44
		Mother’s breastfeeding perceptions	0.30	0.32	**0.021**	0.36
		Mother’s age	−0.67	−0.34	**0.014**	−0.38
		NICU stay ^yes^	−0.08	−0.23	0.090	−0.27
		Father’s formal education	0.84	0.24	0.051	0.31
		Mother–Child dyads	0.18	0.24	0.060	0.30
	8-month model: R = 0.63, Adjusted R^2^ = 0.35, Durbin Watson = 2.42, ANOVA F(3,42) = 9.27, *p* < **0.001**					
	Predictors	NICU stay ^days^	−0.12	−0.35	**0.009**	−0.39
		DBP ^yes^	−11.97	−0.33	**0.009**	−0.39
		Mother–Child dyads	0.11	0.23	0.085	0.26
	12-month model: R = 0.63, Adjusted R^2^ = 0.35, Durbin Watson = 2.01, ANOVA F(3,41) = 9.05, *p* < **0.001**					
	Predictors	Periventricular hemorrhage ^I–IV^	−4.99	−0.40	**0.004**	−0.43
		Mother–Child dyads	0.18	0.28	**0.039**	0.32
		Siblings ^number^	2.40	0.23	0.068	0.28
	18-month model: R = 0.70, Adjusted R^2^ = 0.44, Durbin Watson = 2.31, ANOVA F(4,41) = 9.73, *p* < **0.001**					
	Predictors	NICU stay ^yes^	−0.14	−0.34	**0.008**	−0.40
		Periventricular hemorrhage ^I–IV^	−2.85	−0.29	**0.026**	−0.34
		Leukomalacia ^yes^	−11.36	−0.27	**0.029**	−0.33
		Mother’s formal education	1.30	0.28	**0.026**	0.34
	24-month model: R = 0.66, Adjusted R^2^ = 0.39, Durbin Watson = 1.96, ANOVA F(4,41) = 8.15, *p* < **0.001**					
	Predictors	Periventricular hemorrhage ^I–IV^	−3.92	−0.31	**0.018**	−0.36
		Mother–Child dyads	0.24	0.34	**0.006**	0.41
		Mother’s breastfeeding perception	0.35	0.26	**0.042**	0.31
		Mother’s formal education	1.72	0.29	**0.020**	0.36
**Formula**	4-month model: R = 0.50, Adjusted R^2^ = 0.17, Durbin Watson = 2.13, ANOVA F(4,39) = 3.28, *p* = **0.021**					
	Predictors	Mother’s age	−1.43	−0.56	**0.014**	−0.38
		Father’s age	1.51	0.53	**0.014**	0.38
		Mother–Child dyads	0.23	0.33	**0.028**	0.34
		Siblings ^number^	3.29	0.32	0.055	0.30
	8-month model: R = 0.41, Adjusted R^2^ = 0.13, Durbin Watson = 2.33, ANOVA F(2,41) = 4.25, *p* = **0.021**					
	Predictors	Mother’s formal education	−1.28	−0.33	**0.026**	−0.34
		Periventricular hemorrhage ^I–IV^	−4.29	−0.28	0.058	−0.29
	12-month model: R = 0.55, Adjusted R^2^ = 0.26, Durbin Watson = 2.09, ANOVA F(2,41) = 8.76, *p* < **0.001**					
	Predictors	Mechanical ventilation ^yes^	−0.53	−0.47	**<0.001**	−0.49
		Leukomalacia ^yes^	−17.17	−0.27	**0.042**	−0.31
	18-month model: R = 0.50, Adjusted R^2^ = 0.22, Durbin Watson = 1.77, ANOVA F(2,41) = 6.98, *p* = **0.002**					
	Predictors	Mother–Child dyads	0.41	0.45	**0.002**	0.46
		Periventricular hemorrhage ^I–IV^	−3.57	−0.24	0.084	−0.27
	24-month model: R = 0.64, Adjusted R^2^ = 0.34, Durbin Watson = 1.93, ANOVA F(4390) = 6.61, *p* < **0.001**					
	Predictors	Mother–Child dyads	0.33	0.43	**0.002**	0.48
		DBP ^yes^	−22.59	−0.39	**0.004**	−0.44
		Periventricular hemorrhage ^I–IV^	−4.69	−0.23	0.065	−0.29
		Siblings ^number^	2.39	0.23	0.081	0.27

Note. Units of measurement: “Yes” indicates children with bronchopulmonary dysplasia (BPD); I–IV indicate periventricular hemorrhage grades; number indicates number of siblings at home; and days indicates days on mechanical ventilation. Significant results are presented in bold.

**Table 7:** Multivariate linear regression by groups: language development outcome.

Language	Linear Regression Analysis: Formula Group/Longitudinal by Months		Unstandardized B	Standardized B	*p*	Partial Correlation
**Exclusive breastfeeding**	4-month model: R = 0.41, Adjusted R^2^ = 0.13, Durbin Watson = 1.68, ANOVA F(1,24) = 4.84, *p* = **0.038**					
	Predictor	NICU stay ^days^	−0.16	−0.41	**0.038**	−0.41
	8-month model: R = 0.38, Adjusted R^2^ = 0.11, Durbin Watson = 1.80, ANOVA F(1,24) = 3.98, *p* = **0.050**					
	Predictor	Family income	−0.01	−0.38	**0.050**	−0.38
	12-month model: R = 0.57, Adjusted R^2^ = 0.23, Durbin Watson = 1.85, ANOVA F(3,22) = 3.55, *p* = **0.031**					
	Predictors	Mother’s formal education	3.34	0.39	**0.046**	0.37
		Father’s age	1.15	0.32	0.091	0.31
		DBP ^yes^	−18.03	−0.33	0.082	−0.32
	18-month model: R = 0.62, Adjusted R^2^ = 0.33, Durbin Watson = 2.27, ANOVA F(2,23) = 7.25, *p* = **0.004**					
	Predictors	Mother–Child dyads	0.56	0.56	**0.002**	0.56
		Father’s age	1.12	0.35	**0.046**	0.34
	24-month model: R = 0.15, Adjusted R^2^ = 0.02, Durbin Watson = --, ANOVA F(1,24) = 0.52, *p* = 0.478					
	Predictor	Siblings ^number^	0.64	0.15	0.478	0.15
**Mixed** **feeding**	4-month model: R = 0.77, Adjusted R^2^ = 0.59, Durbin Watson = 2.16, ANOVA F(6,39) = 9.57, *p* < **0.001**					
	Predictors	Father’s formal education	1.89	0.45	**<0.001**	0.57
		Mother–Child dyads	0.39	0.45	**<0.001**	0.56
		Leukomalacia ^yes^	−9.71	−0.22	**0.048**	−0.31
		Breastfeeding length	0.27	0.24	**0.030**	0.34
		Mother’s age	−0.54	−0.23	**0.031**	−0.34
		NICU stay ^days^	−0.08	−0.19	0.069	−0.29
	8-month model: R = 0.45, Adjusted R^2^ = 0.18, Durbin Watson = 2.50, ANOVA F(1,44) = 11.12, *p* = **0.002**					
	Predictor	Mother–Child dyads	0.33	0.45	**0.002**	0.45
	12-month model: R = 0.36, Adjusted R^2^ = 0.11, Durbin Watson = 1.70, ANOVA F(1,43) = 6.63, *p* = **0.014**					
	Predictor	Mechanical ventilation ^days^	−0.35	−0.36	**0.014**	−0.36
	18-month model: R = 0.49, Adjusted R^2^ = 0.22, Durbin Watson = 2.09, ANOVA F(1,44) = 13.73, *p* < **0.001**					
	Predictor	NICVU stay ^days^	−0.19	−0.49	**<0.001**	−0.49
	24-month model: R = 0.56, Adjusted R^2^ = 0.28, Durbin Watson = 2.13, ANOVA F(2,43) = 9.90, *p* < **0.001**					
	Predictors	Mother’s breastfeeding perceptions	0.45	0.39	**0.004**	0.42
		Mother–Child dyads	0.22	0.36	**0.007**	0.40
**Formula**	4-month model: R = 0.50, Adjusted R^2^ = 0.18, Durbin Watson = 2.50, ANOVA F(1,39) = 3.32, *p* = **0.020**					
	Predictors	Father’s age	1.21	0.51	**0.019**	0.36
		Mother’s age	−1.06	−0.50	**0.025**	−0.35
		Mother’s formal education	1.42	0.34	**0.025**	0.35
		NICU stay ^days^	−0.11	−0.26	0.087	−0.27
	8-month model: R = 0.41, Adjusted R^2^ = 0.13, Durbin Watson = 2.35, ANOVA F(2,41) = 4.28, *p* = **0.021**					
	Predictors	DBP ^yes^	−10.28	−0.31	**0.032**	−0.33
		Father’s age	−0.76	−0.29	**0.045**	−0.27
	12-month model: R = 0.42, Adjusted R^2^ = 0.14, Durbin Watson = 2.50, ANOVA F(2,41) = 4.48, *p* = **0.017**					
	Predictors	Mother–Child dyads	0.18	0.34	**0.021**	0.35
		DBP ^yes^	−9.43	−0.30	**0.041**	−0.25
	18-month model: R = 0.49, Adjusted R^2^ = 0.20, Durbin Watson = 1.72, ANOVA F(2,41) = 6.49, *p* = **0.004**					
	Predictors	Mother’s age	−0.72	−0.55	**<0.001**	−0.49
		Siblings ^number^	1.68	0.32	**0.045**	0.31
	24-month model: R = 0.59, Adjusted R^2^ = 0.31, Durbin Watson = 1.56, ANOVA F(2,41) = 10.86, *p* < **0.001**					
	Predictors	Mother–Child dyads	0.36	0.54	**<0.001**	0.56
		Mechanical ventilation ^yes^	−0.23	−0.25	0.056	−0.29

Note. Units of measurement: “Yes” indicates children with bronchopulmonary dysplasia (BPD); I–IV indicate periventricular hemorrhage grades; number indicates number of siblings at home; and days indicates days on mechanical ventilation. Significant results are presented in bold.

**Table 8:** Multivariate linear regression by groups: social development outcome.

Social	Linear Regression Analysis: Formula Group/Longitudinal by Months		Unstandardized B	Standardized B	*p*	Partial Correlation
**Exclusive breastfeeding**	4-month model: R = 0.98, Adjusted R^2^ = 0.95, Durbin Watson = 1.40, ANOVA F(4,21) = 116.7, *p* < **0.001**					
	Predictors	Mother–Child dyads	0.25	0.99	**<0.001**	0.84
		Father’s age	0.07	0.16	**0.010**	0.13
		BPD ^yes^	−0.71	−0.11	**0.031**	0.10
		Siblings ^yes^	0.19	0.12	0.065	0.09
	8-month model: R = 0.90, Adjusted R^2^ = 0.79, Durbin Watson = 1.14, ANOVA F(3,22) = 31.94, *p* < **0.001**					
	Predictors	Mother–Child dyads	0.22	0.79	**<0.001**	0.77
		BPD ^yes^	−1.56	−0.22	**0.032**	−0.21
		Mother’s formal education	0.19	0.17	0.081	0.17
	12-month model: R = 0.93, Adjusted R^2^ = 0.87, Durbin Watson = 1.95, ANOVA F(1,24) = 165.4, *p* < **0.001**					
	Predictor	Mother–Child dyads	0.24	0.93	**<0.001**	0.93
	18-month model: R = 0.78, Adjusted R^2^ = 0.58, Durbin Watson = 1.75, ANOVA F(2,23) = 18.09, *p* < **0.001**					
	Predictors	Mother–Child dyads	0.25	0.62	**<0.001**	0.60
		BPD ^yes^	−6.44	−0.32	**0.025**	−0.31
	24-month model: R = 0.70, Adjusted R^2^ = 0.45, Durbin Watson = 2.04, ANOVA F(2,23) = 11.23, *p* < **0.001**					
	Predictors	Family income	0.03	0.55	**0.001**	0.55
		BPD ^yes^	−4.62	−0.50	**0.003**	0.55
**Mixed** **feeding**	4-month model: R = 0.92, Adjusted R^2^ = 0.84, Durbin Watson = 1.98, ANOVA F(1,44) = 241.7, *p* < **0.001**					
	Predictor	Mother–Child dyads	0.20	0.92	**<0.001**	0.92
	8-month model: R = 0.88, Adjusted R^2^ = 0.77, Durbin Watson = 1.95, ANOVA F(4,41) = 148.8, *p* < **0.001**					
	Predictor	Mother–Child dyads	0.26	0.88	**<0.001**	0.88
	12-month model: R = 0.85 Adjusted R^2^ = 0.71, Durbin Watson = 1.99, ANOVA F(2,42) = 54.49, *p* < **0.001**					
	Predictors	Mother–Child dyads	0.23	0.698	**<0.001**	0.77
		Mechanical ventilation ^days^	−1.96	−0.29	**0.002**	−0.45
	18-month model: R = 0.93, Adjusted R^2^ = 0.86, Durbin Watson = 1.98, ANOVA F(2,43) = 138.8, *p* < **0.001**					
	Predictors	Mother–Child dyads	0.31	0.93	**<0.001**	0.93
		Breastfeeding length	0.08	0.15	**0.011**	0.37
	24-month model: R = 0.91, Adjusted R^2^ = 0.82, Durbin Watson = 1.92, ANOVA F(3,42) = 69.82, *p* < **0.001**					
	Predictors	Mother–Child dyads	0.27	0.89	**<0.001**	0.90
		NICU stay ^days^	−0.04	−0.17	**0.012**	0.38
		Breastfeeding length	0.09	0.16	**0.019**	0.35
**Formula**	4-month model: R = 0.90, Adjusted R^2^ = 0.80, Durbin Watson = 2.17, ANOVA F(1,42) = 177.7, *p* < **0.001**					
	Predictor	Mother–Child dyads	0.19	0.90	**<0.001**	0.90
	8-month model: R = 0.92, Adjusted R^2^ = 0.84, Durbin Watson = 2.09, ANOVA F(2,41) = 113.5, *p* < **0.001**					
	Predictors	Mother–Child dyads	0.25	0.92	**<0.001**	0.92
		Father’s age	−0.12	−0.11	0.072	−0.28
	12-month model: R = 0.88, Adjusted R^2^ = 0.76, Durbin Watson = 1.88, ANOVA F(1,42) = 138.4, *p* < **0.001**					
	Predictor	Mother–Child dyads	0.27	0.88	**<0.001**	0.88
	18-month model: R = 0.84, Adjusted R^2^ = 0.69, Durbin Watson = 2.16, ANOVA F(2,41) = 47.95, *p* < **0.001**					
	Predictors	Mother–Child dyads	0.22	0.78	**<0.001**	0.81
		Mother’s age	−0.10	−0.16	0.070	−28
	24-month model: R = 0.90, Adjusted R^2^ = 0.80, Durbin Watson = 1.52, ANOVA F(1,42) = 173.6, *p* < **0.001**					
	Predictor	Mother–Child dyads	0.29	0.90	**<0.001**	0.90

Note. Units of measurement: “Yes” indicates children with bronchopulmonary dysplasia (BPD); I–IV indicate periventricular hemorrhage grades; number indicates number of siblings at home; and days indicates days on mechanical ventilation. Significant results are presented in bold.

## Data Availability

The data are available upon request from the authors.
